# Washed microbiota transplantation: candidates for a novel strategy for ameliorating autism spectrum disorder

**DOI:** 10.3389/fmicb.2025.1688325

**Published:** 2025-11-11

**Authors:** Shuo Feng, Jiangyan Wang, Xinyu Si, Shenghua Lu, Caimei Lu, Zheng Gao, Juan Yang, Jiali Wu, Xingxiang He, Lei Wu

**Affiliations:** 1 Department of Gastroenterology, Research Center for Engineering Techniques of Microbiota-Targeted Therapies of Guangdong Province, The First Affiliated Hospital of Guangdong Pharmaceutical University, Guangzhou, China; 2 Guangdong Provincial Key Laboratory for Research and Evaluation of Pharmaceutical Preparations, Guangzhou, China; 3 Guangdong Pharmaceutical University, Guangzhou, China

**Keywords:** autism spectrum disorder (ASD), gut microbiota, washed microbiota transplantation (WMT), fecal microbiota transplantation (FMT), therapeutic strategies

## Abstract

Autism Spectrum Disorder (ASD) is a severe neurodevelopmental disorder with an increasing global incidence, imposing substantial burdens on both society and affected families. The pathogenesis of ASD is complex, involving genetic, environmental, and other factors. Notably, children with ASD often exhibit gut microbiota dysbiosis, and the relationship between gut microbiota and ASD has garnered growing attention. Current treatments for ASD remain limited and unsatisfactory. As an emerging therapeutic approach, Washed Microbiota Transplantation (WMT) reduces undigested food residues, fungi, parasite eggs, and pro-inflammatory metabolites, thereby lowering the incidence of adverse clinical events. WMT also addresses ethical and aesthetic concerns associated with Fecal Microbiota Transplantation (FMT), enhances treatment safety, and offers new hope for ASD management. This review integrates global literature to analyze the latest findings on ASD epidemiology, societal impacts, existing therapies, and clinical research on WMT, aiming to provide scientific evidence for the clinical application of WMT in ASD treatment.

## 1 Introduction

Autism Spectrum Disorder (ASD) is a complex neurodevelopmental condition characterized by social communication deficits and restricted, repetitive patterns of behavior (American Psychiatric Association, 2022). Since Kanner’s first clinical description in 1943, ASD has gained global recognition (Kanner, 1943). Recent studies report an ASD prevalence of 0.7% in China, with over 10 million affected individuals and an annual increase of nearly 200,000 cases (Report on the Development Status of China's Autism Education and Rehabilitation Industry, 2024). According to the U.S. Centers for Disease Control and Prevention (2020), the childhood ASD prevalence in the U.S. rose from 1 in 44 in 2018 to 1 in 36 by 2020, while global estimates range between 2.3% and 2.76% (Hirota and King, 2023; Maenner et al., 2021; Maenner et al., 2023). The escalating prevalence of ASD has positioned it as a critical public health challenge worldwide.

Core symptoms of ASD include persistent deficits in social communication across multiple contexts and restricted, repetitive behaviors or interests (Zwaigenbaum and Penner, 2018). Beyond neurological abnormalities, ASD patients frequently present with gastrointestinal (GI) symptoms such as abdominal pain, bloating, diarrhea, constipation, and flatulence. These GI disturbances strongly correlate with ASD severity (Johnson et al., 2020). The gut, a microbial ecosystem, harbors diverse microbiota that interact closely with the host. Emerging evidence highlights the pivotal role of gut microbiota in regulating neurological functions and central nervous system (CNS)-associated behaviors (Johnson et al., 2020). Although the pathogenesis of ASD involves multifactorial interactions, including genetic and environmental triggers (Han et al., 2021), therapeutic options remain limited, underscoring the urgent need for effective interventions. Growing clinical evidence links ASD to gut microbiota dysbiosis, marked by reduced microbial diversity, diminished beneficial bacteria, and altered microbial composition in affected children (Dan et al., 2020; Wang M. et al., 2019). Fecal Microbiota Transplantation (FMT), a strategy to reconstitute gut microbiota, has emerged as a promising therapeutic avenue for ASD (Sharon et al., 2019). Washed Microbiota Transplantation (WMT), an advanced iteration of FMT, employs intelligent fecal microbiota processing systems to isolate, purify, and administer microbiota from healthy donors. By systematically removing harmful components through repeated washing, WMT improves safety, minimizes adverse effects, and ensures quality control compared to conventional FMT (Zhang et al., 2020). This review synthesizes current advancements in WMT for ASD treatment, aiming to inspire novel research directions and clinical applications.

## 2 The impact of ASD

### 2.1 Effects on patients

Individuals with ASD typically face challenges in social interaction and communication, which negatively affect their ability to initiate and sustain relationships (Chevallier et al., 2012). Restricted, repetitive behavioral patterns and narrow interests may limit daily functioning and learning capabilities, while impulsive behaviors further compromise quality of life (Wang et al., 2021). Differences in executive functioning and local processing abilities are common, impairing performance in theory of mind tasks (Demetriou et al., 2018). Comorbid conditions such as epilepsy, attention deficits, gastrointestinal disorders, oppositional behaviors, anxiety, depression, sleep disturbances, and eating disorders exacerbate the physical, emotional, and financial burden on patients and families (Kong and Chen, 2023). Additionally, social exclusion and stigmatization negatively impact self-identity, rehabilitation, and mental well-being (Kong and Chen, 2023).

### 2.2 Effects on families

The long-term costs of specialized education, rehabilitation therapies (e.g., speech training, behavioral interventions), and adaptive equipment impose significant financial strain. In the UK, lifetime costs for caring for a child with ASD exceed £920,000, while in the U. S. and China, these costs reach $1.4 million and ¥116,134.44, respectively (Buescher et al., n.d.; Zhao et al., 2021b). Behavioral challenges and communication barriers often lead to parental frustration, helplessness, and anxiety, with prolonged caregiving contributing to psychological fatigue and emotional burnout (Lee et al., 2024; Wu et al., 2024). Marital conflicts may arise due to disagreements over caregiving approaches and financial pressures, increasing divorce rates and straining sibling relationships (Karst and Van Hecke, 2012; Hartley et al., 2010; Ilias et al., 2019). Social stigma and embarrassment over atypical behaviors in public settings isolate families, reducing social engagement and access to support networks (Wu et al., 2024; Ilias et al., 2019; Li and Wang, 2015). Caregivers frequently sacrifice personal goals, career opportunities, and leisure activities, with one parent often leaving the workforce to provide full-time care, further impacting household income (Wu et al., 2024; Ilias et al., 2019). Persistent guilt and self-blame for their child’s condition also undermine parental mental health (Wu et al., 2024; Wong et al., 2015).

### 2.3 Societal implications

The rising prevalence of ASD places substantial economic burdens on families and society, including medical expenses, special education costs, and lost productivity due to caregiver absenteeism (Wu et al., 2024; Ilias et al., 2019). Adults with ASD often struggle with employment, perpetuating socioeconomic challenges (Fu and Chen, 2023). Educational systems face pressure to provide tailored environments and interventions to support ASD students’ learning and social integration. Public health infrastructure must prioritize early diagnosis, lifelong intervention services, and equitable access to care. Social participation and acceptance remain hindered by stigma and misconceptions, necessitating societal awareness campaigns and inclusive policies (Wu et al., 2024; Lan and Bai, 2020).

Key Recommendations: Public Awareness: Combat stigma through education to foster societal inclusion. Early Intervention: Strengthen diagnostic and therapeutic services to improve outcomes. Multidisciplinary Collaboration: Integrate medical, psychological, and educational expertise for holistic care. Policy Development: Implement public health strategies addressing prevention, early diagnosis, and lifelong support. Research Investment: Prioritize studies on ASD etiology, mechanisms, and innovative therapies.

## 3 Current treatment approaches for ASD and their limitations

### 3.1 Behavioral interventions

Early intervention is critical for ASD due to its early onset and better outcomes with younger age. Behavioral interventions remain a cornerstone of ASD management, with key methods and their characteristics as follows (see Table 1 for details):

**Table 1:** Comparison of the main intervention methods.

Intervention	Advantages	Limitations
ABA	High practicality; improves cognition, communication, and language skills (Lovaas, 1987)	Limited peer interaction opportunities; minimal impact on social deficits; requires costly cross-cultural training; difficult to integrate into mainstream education (Ding et al., 2015; Wang Y. et al., 2019)
SCERTS	Flexible for various social situations, avoiding the generalization problem of traditional one – on – one training (Yang, 2007)	Higher demands on families and schools; controversial in assessment indicator selection and ecological validity (Tan et al., 2021)
EI	Improves patients’ symptoms, behaviors and quality of life, with better effects from medium – to – high – intensity exercise than low – intensity exercise (Bremer et al., 2016; Liang et al., 2022; Codella et al., 2018)	Requires adult accompaniment and professional coach guidance to prevent sports injuries; some children’s tactile sensitivity may lead to resistance against wearing heart rate monitors, causing aversion and non - compliance (Zhao et al., 2021a)
PT	Improves children’s social interaction, attention, emotional behavior and motor skills, especially daily living skills (Tan et al., 2021)	Children may struggle with game rules, have low task completion rates, exhibit destructive behaviors, face limited therapy settings, lack responsiveness during treatment, and encounter generalization and waning interest issues (Tan et al., 2021; Xu et al., 2019)
MT	Enhances children’s emotional regulation, social skills, joint attention, imitation and peer interaction (Geretsegger et al., 2014)	Restricted participation by non - professionals; shortage of professional music therapists; high intervention costs; low parental acceptance (Geretsegger et al., 2014)
SIT	Benefits children’s language, social, sensory, and behavioral aspects, and improves physical fitness (Tan et al., 2021)	Incomplete theoretical basis; monotonous teaching methods; time - consuming and labor - intensive intervention process; increases family financial burden (Tan et al., 2021; Zhao et al., 2017)
VMI	Effective in boosting children’s academic, adaptive, social communication and vocational skills; offers immediate feedback; cost – effective (Xiao et al., 2013)	Needs improvement in video design, editing, and ensuring attention and learning outcomes (Xiao et al., 2013; Syriopoulou-Delli and Sarri, 2021)

ABA: Applied Behavior Analysis; SCERTS: Social Communication, Emotional Regulation, and Transactional Support; EI: Exercise Intervention; PT: Play Therapy; MT: Music Therapy; SIT: Sensory Integrative Training; VMI: Video Modeling Intervention.

ABA: High practicality in improving cognition and language, but limited in social deficit improvement and high cross-cultural training costs (Lovaas, 1987; Ding et al., 2015; Wang Y. et al., 2019).

SCERTS: Flexible for social scenarios but demands high on families/schools (Yang, 2007; Tan et al., 2021).

EI: Medium-to-high intensity exercise better improves symptoms but requires professional guidance (Bremer et al., 2016; Liang et al., 2022; Zhao et al., 2021a).

PT/MT/SIT/VMI: Each has advantages in specific domains (e.g., MT for emotional regulation, VMI for cost-effectiveness) but faces limitations like limited settings or professional shortages (Tan et al., 2021; Geretsegger et al., 2014; Xiao et al., 2013; Xu et al., 2019; Zhao et al., 2017; Syriopoulou-Delli and Sarri, 2021).

Overall, behavioral interventions require long-term evaluation, and their accessibility is restricted by resource scarcity and cost, highlighting the need for complementary strategies.

### 3.2 Pharmacological interventions

Drug treatment mainly targets comorbidiasis symptoms of ASD. For instance, risperidone and aripiprazole have been approved by the FDA for the treatment of irritability symptoms related to ASD (Stepanova et al., 2017; Shafiq and Pringsheim, 2018). However, there is currently no drug that can effectively improve the core symptoms of ASD, and long-term use may cause significant side effects, limiting their clinical application (Sharma et al., 2018; Stepanova et al., 2017; Shafiq and Pringsheim, 2018; Persico et al., 2021; Yan et al., 2022; Kanner and Bicchi, 2022; Alvares et al., 2017; Lemonnier et al., 2017; Howes et al., 2018; Ming et al., 2008; Elchaar et al., 2006; Malow et al., 2021; Xu and Wang, 2018) (see Table 2 for details).

**Table 2:** Comparison of common pharmacotherapies.

Drug class	Examples	Advantages	Limitations
Atypical antipsychotics	Risperidone, Aripiprazole (FDA-approved for ASD-associated irritability)	Reduce irritability and aggressive behaviors in ASD children	Side effects: extrapyramidal symptoms, sedation, weight gain; risk of tolerance and dependence with prolonged use
	Olanzapine, Quetiapine, Ziprasidone, Lurasidone	Manage irritability	Limited trial data; greater side effects; efficacy and safety require validation
Antidepressants	Fluoxetine, Sertraline, Citalopram, Escitalopram, Fluvoxamine	Alleviate depressive symptoms and improve mood	Side effects: sedation, agitation, insomnia, appetite loss, attention deficits, hyperactivity, urinary retention
Antiepileptics	Sodium valproate, Levetiracetam, Lamotrigine	Reduce repetitive behaviors, irritability, and hyperactivity	Minimal benefit for core ASD symptoms; side effects (agitation, insomnia); clinical value unconfirmed
CNS Stimulants	Methylphenidate, Amphetamines	Mitigate attention deficits and hyperactivity	Side effects: insomnia, appetite suppression, cardiovascular risks
Investigational agents	Oxytocin	Great potential in improving social disorders in children with ASD	Inconsistent efficacy for core symptoms; requires further validation
	Bumetanide	May ameliorate core ASD symptoms	Adverse events: hypokalemia, polyuria, appetite loss, dehydration, fatigue
	Memantine	Improves language, social behaviors, and self-stimulation; long-term safety	Short-term trials show limited efficacy; better suited for combination therapy
	Clonidine	Reduces hyperactivity, aggression, and mood swings	Side effects: insomnia, headaches
	Naltrexone	Effective for self-injury, hyperactivity, and stereotypy	Sedation, compliance challenges in trials
	Melatonin	Improves sleep quality with good tolerability	Mild side effects: fatigue, drowsiness

### 3.3 Traditional Chinese medicine (TCM) approaches

TCM attributes ASD to brain dysfunction linked to kidney/spleen/liver/heart imbalances, with interventions including herbal medicine, acupuncture, and tuina. Limitations include low pediatric compliance (bitter herbs, needle phobia) and insufficient large-scale trials (Yan et al., 2022; Zhang et al., 2023). Notably, TCM’s historical use of “Jin Zhi” (golden juice, a fecal-based medicine) represents an early precursor to modern microbiota transplantation (Shi and Yang, 2017; Zhang et al., 2012; Xu et al., 2017), aligning with the gut-brain axis theory and providing a historical context for WMT’s development.

## 4 Research progress on WMT for ASD

Autism Spectrum Disorder (ASD) arises from complex interactions between genetic and environmental factors, with gut microbiota dysbiosis emerging as a key environmental contributor. The human gut hosts 10–100 trillion microorganisms, including bacteria, viruses, protozoa, and fungi, collectively termed the gut microbiota. ASD patients exhibit gut dysbiosis characterized by pathogenic overgrowth and metabolic disturbances. Studies report that 9–91% of ASD patients experience gastrointestinal (GI) symptoms such as bloating, diarrhea, constipation, and foul-smelling stools (Moradi et al., 2021; Holingue et al., 2018), often accompanied by esophagitis, gastritis, or lymphocytic infiltration, with approximately half suffering from alternating diarrhea and constipation (Duan et al., 2015). Notably, alleviating GI symptoms correlates with reduced ASD severity (Chen and Chiu, 2022). Recent evidence underscores the role of gut microbiota in ASD pathogenesis, with interventions like probiotics and Fecal Microbiota Transplantation (FMT) showing therapeutic potential (Strati et al., 2017; Kang et al., 2017; Kang et al., 2019). Unlike probiotics, which deliver single bacterial strains, FMT introduces ~1,000 native gut bacterial species, almost full coverage of all strains has been achieved. However, Washed Microbiota Transplantation (WMT), an advanced FMT technique, enhances safety by removing undigested residues, fungi, parasite eggs, and pro-inflammatory metabolites, thereby significantly reducing the incidence of clinical adverse reaction events, while addressing ethical and aesthetic concerns associated with traditional FMT (Zhang et al., 2020; Shi, 2020).

### 4.1 Principles of WMT

WMT a new stage in the development of FMT involves transferring fecal microbiota from healthy donors into a patient’s gastrointestinal tract through various methods, aiming to reconstruct the patient’s gut microbiota and thereby treat intestinal and systemic diseases. The human microbiota refers to the collective community of microorganisms residing on and within the human body, evolving from the concept of “normal human flora.” These microbial communities are most densely distributed in mucosal organs, such as the oral cavity and intestines (Integrative HMP (iHMP) Research Network Consortium, 2019; Lloyd-Price et al., 2016). The gut microbiota, often termed the “second genome” of the human body, harbors vast numbers of microorganisms, including bacteria, fungi, and others. Studies estimate that approximately 70% of the human microbiota resides in the colon (Ley et al., 2006), with significant individual variations. Research has linked these variations to differences in human health and disease manifestations (Garcia et al., 2022). Gut microbiota plays a critical role in host health, contributing to nutrient absorption, metabolic regulation, intestinal epithelial development, and the induction of innate immunity. Its functions are comparable to those of a vital organ. By transplanting healthy gut microbiota, the intestinal microecology of patients can be modulated to improve immune and neurological functions, thereby achieving therapeutic goals for various diseases.

### 4.2 Preparation and administration methods of fecal material

The entire process—from the acquisition and preparation of fecal microbiota to its safe delivery and functional integration into the intestines—requires stringent control. Efforts focus on identifying optimal fecal donors, developing preservation methods that maximize therapeutic benefits for patients, and selecting the most suitable administration routes to ensure the microbiota exerts its full effect post-transplantation. For WMT, fecal material is primarily sourced from rigorously screened healthy donors. Donors undergo comprehensive evaluations, including gastrointestinal pathogen screening, blood tests, and mental health assessments, to ensure the fecal material does not transmit infectious diseases to recipients (Shi, 2020). Subsequently, in high-standard laboratory settings, the fecal microbiota is isolated using an intelligent fecal microbiota separation system. This involves steps such as hydration, homogenization, filtration, centrifugation, and sedimentation to obtain purified bacterial preparations (Shi, 2020). Purified microbiota can be preserved via freezing, refrigeration, or specialized storage solutions for sample collection, storage, and transportation under ambient conditions (Shi, 2020). Microcrystalline cellulose particles, a free-flowing material, are employed as adsorbents to concentrate and filter fresh fecal samples. This method maintains microbial viability and diversity even in dry environments. Clinically common WMT administration routes target the upper or lower gastrointestinal tract. Methods include delivery via nasogastric/nasojejunal tubes, oral capsules, esophagogastroduodenoscopy, colonoscopy, enema, or endoscopic techniques (e.g., transendoscopic enteral tubing or percutaneous endoscopic cecostomy). The optimal method is selected based on the patient’s tolerance, disease location, and clinical characteristics.

### 4.3 Clinical research on WMT for ASD

Currently, FMT is not only used to treat gastrointestinal diseases such as inflammatory bowel disease, irritable bowel syndrome, functional constipation, and cirrhosis but has also been applied to neuropsychiatric disorders (e.g., ASD, anxiety, depression, and Parkinson’s disease), metabolic diseases (e.g., diabetes, obesity, fatty liver, and hyperlipidemia), and immune-related conditions (e.g., cancer immunotherapy, allergic diseases, and chronic fatigue syndrome), demonstrating promising clinical efficacy (Ye et al., 2020). WMT traces its origins to traditional Chinese medicine (TCM). The earliest documented use of human feces as medicine dates back to Wushier Bingfang (Fifty-Two Disease Formulas), a medical text from the Western Zhou or Spring and Autumn period (Shi and Yang, 2017). Ge Hong’s Zhouhou Beiji Fang (Handbook of Prescriptions for Emergencies) from the Eastern Jin Dynasty records the therapeutic use of fecal solutions: “Drinking one liter of fecal juice revives the patient” (Zhang et al., 2012). Li Shizhen’s Compendium of Materia Medica (Ben Cao Gang Mu) from the Ming Dynasty describes over 20 medicinal formulations utilizing human feces. During the Ming and Qing dynasties, fecal-based treatments became widely employed for epidemic febrile diseases. Notably, the physician Ye Tianshi documented cases using fecal preparations to treat such conditions. As febrile diseases often involved heat toxins affecting the gastrointestinal tract, Jin Zhi (golden juice) was frequently used with remarkable efficacy. Its preparation involved collecting feces from healthy boys aged 11–12 during winter, mixing it with mountain spring or well water, filtering, and burying the mixture in a sealed jar for over a year. The resulting clear, water-like liquid was termed Jin Zhi (Xu et al., 2017). In modern times, the use of Jin Zhi declined due to the diversification of TCM practices and the introduction of Western medicine. However, in 2012, Professor Zhang Faming from the Second Affiliated Hospital of Nanjing Medical University pioneered standardized modern fecal microbiota transplantation in China. Using advanced laboratory equipment, his team developed a refined method to isolate highly purified microbiota (now termed WMT), which is regarded as the “new Jin Zhi” (Shi, 2020).

As an emerging therapeutic approach, WMT has garnered significant attention in recent years for treating autism spectrum disorder (ASD), with a growing body of clinical studies specifically investigating its use. These studies, summarized in Table 3, suggest that WMT can alleviate core behavioral symptoms, gastrointestinal distress, and comorbidities in autistic patients (Kang et al., 2017; Kang et al., 2019; Li et al., 2019; Zhao et al., 2017; Luo, 2018; Qian, 2024; Li et al., 2021; Liu et al., 2024; Chen et al., 2022c; Chen et al., 2022a; Chen et al., 2020; Zhao et al., n.d.; Wei et al., 2023; Li et al., 2024a; Chen et al., 2022b; Hu et al., 2023; Wang et al., 2024; Hazan et al., 2024; Qureshi et al., 2020; Zhang et al., 2022; Liu et al., 2023; Pan et al., 2022; Li et al., 2022; Zeng et al., 2023; Ward et al., 2016; Li et al., 2024b; Hu et al., 2025) (see Table 3). Multiple studies on the efficacy and safety of WMT have shown that, multiple studies demonstrate that WMT significantly improves autism-like behaviors, sleep disturbances, stool consistency, gastrointestinal symptoms, and systemic inflammation in ASD patients (Pan et al., 2022). Concurrently, WMT shifts the gut microbiota and metabolic profiles of ASD patients toward beneficial directions. Research methodologies have evolved from open-label designs to multicenter, randomized, double-blind, placebo-controlled trials, enhancing rigor and comprehensiveness. For instance at Peking Union Medical College Hospital, over 100 children with ASD showed marked improvement following FMT-based therapy, with an efficacy rate nearing 80% (Li et al., 2022), and no severe complications were observed during treatment. In terms of the impact of WMT on the Gut Microbiota, Post-WMT, ASD patients exhibit increased gut microbiota diversity, aligning closer to donor profiles (Kang et al., 2017; Kang et al., 2019; Hu et al., 2023). Notably, FMT elevates the relative abundance of Bifidobacterium and Prevotella in the gut, both of which may confer therapeutic benefits for ASD (Kang et al., 2017; Kang et al., 2019). Long-Term Efficacy, studies indicate sustained effects of FMT for up to 2 years. Kang et al. (2019) reported persistent improvements in ASD and gastrointestinal symptoms over a two-year follow-up. Li Ning et al. followed 85 ASD cases for 3 years, observing a 78.8% response rate at 6 months, 71.9% at 1 year, and 60% efficacy at 3 years, with over 20% achieving clinical cure (Li et al., 2019). Chen et al. (2022c) and Chen et al. (2022a) documented maintained therapeutic effects on ABC (Autism Behavior Checklist) and BSFS (Bristol Stool Form Scale) scores for 48 months, with results comparable to initial outcomes at 60 months. Chen et al. (2020) reported efficacy in 7 out of 12 ASD patients during a 60-month follow-up. Among the adverse events of WMT, most adverse events during WMT are mild to moderate, including abdominal distension, nausea, vomiting, diarrhea, fever, and hyperactivity. These resolved with temporary treatment suspension or symptomatic management, with no severe adverse events reported. Intriguingly, Ward et al. (2016) found WMT more effective in younger ASD patients, while older children showed limited responses. Family donor microbiota may play a critical role in outcomes for older patients (Hazan et al., 2024), warranting further exploration. This age-dependent efficacy may stem from the immature gut microbiota structure in younger individuals, which is more susceptible to external modulation. Studies highlight enhanced efficacy when WMT is combined with routine rehabilitation training (RAU), surpassing RAU alone in alleviating clinical and gastrointestinal symptoms (Qian, 2024; Zeng et al., 2023). This underscores WMT’s potential to restore gut microbial balance and ameliorate ASD manifestations. Additionally, WMT demonstrated superior efficacy compared to probiotic treatments in ASD children (Liu et al., 2024). In terms of metabolite changes in WMT, Post-WMT, plasma metabolite profiles in ASD children align more closely with those of neurotypical children (Qureshi et al., 2020). WMT also significantly reduces urinary 5-HIAA (5-hydroxyindoleacetic acid) levels (Wang et al., 2024), regulate the intestinal flora by reducing toxic metabolites and enhancing detoxification effects (Liu et al., 2023), suggesting positive impacts on metabolic health. In terms of the WMT treatment course, it was found that WMT could improve ASD symptoms, gastrointestinal symptoms and sleep disorders, and multiple treatments could bring more significant improvements (Pan et al., 2022). Six treatments could bring significant improvements, and patients with gastrointestinal symptoms before treatment had better therapeutic effects (Hu et al., 2025), indicating that the number of WMT treatment courses also has a positive effect on children with ASD.

**Table 3:** Clinical Studies on FMT or WMT in ASD.

Citation	Study design	Gut intervention	Protocol (FMT/WMT)	Duration	Behavioral outcomes	Gut outcomes	Microbial outcomes	Other outcomes	Adverse events
Kang et al. (2017), USA	Open-label; FMT: 18 ASD children (ages 7–16y) with moderate to severe GI symptoms; Control: 20 age/gender-matched neurotypical children without GI symptoms	Oral vancomycin and acid pump inhibitor; bowel cleansing	FMT: 12 oral (SHGM): 2.5 × 10^12^ cells/d for 2d → 2.5 × 10^9^ cells/d for 8w; 6 rectal (enema): single dose of 2.5 × 10^12^ cells → 2.5 × 10^9^ cells/d orally for 7w	8 weeks	CARS: 24% reduction; PGI-III, ABC, SRS, VABS-II: improved	GSRS: 77% reduction; DSR: improved	α-diversity ↑; β-diversity shifted toward donor; ↑ *Bifidobacterium*, *Prevotella*, *Desulfovibrio*	N/A	Vancomycin: 28–39% mild hyperactivity/tantrums; Oral SHGM: 5% nausea
Kang et al. (2019), USA	Open-label; Same cohort as (Kang et al., 2017)	Same as (Kang et al., 2017)	FMT: Same as (Kang et al., 2017)	2 years post-FMT	CARS: 47% reduction; PGI-III, ABC, SRS, VABS-II: maintained improvement	GSRS: 58% reduction; DSR: sustained improvement	α-diversity ↑; β-diversity similar to pre-FMT; ↑ *Bifidobacterium*, *Prevotella*	N/A	N/A
Li et al. (2019), China	Case series; FMT: 85 ASD children with constipation/diarrhea	Nasogastric polyethylene glycol until watery stool	Oral FMT capsules: 100 g feces → 500 mL saline → filtered → freeze-dried capsules; 6-day oral course	3 years post-FMT	N/A	N/A	N/A	Clinical cure rates: 23.5% (3 m), 22.8% (12 m), 20.0% (36 m); Improvement rates: 55.3% (3 m), 49.1% (12 m), 40.0% (36 m)	Abdominal discomfort (7.1%), nausea/vomiting (9.4%), transient fever (1.2%), diarrhea (3.5%); resolved within 1–3 days
Zhao et al. (2017), China	Case report; FMT: 1 ASD child (age 8y)	20 mL mannitol + 500 mL warm saline enema	FMT: 400 mL donor fecal suspension (single dose)	8 weeks	CARS: 15.7% reduction; ABC improved	Hiccups resolved	N/A	Improved language, communication, sleep, and emotional regulation	N/A
Luo (2018), China	41 ASD (ages 3–7y) vs. 37 controls; FMT: 4 ASD	Mannitol enema	FMT: 80–100 g feces → 500 mL saline → 400 mL single dose	2 months	ABC: 25% reduction	N/A	↑ Bifidobacterium, Acidaminococcus, Catenibacterium, Lactococcus, Megasphaera; ↓ Firmicutes/Bacteroidetes ratio	Improved sleep in 1 case	1 fever, 1 vomiting; resolved
Qian (2024), China	RCT; 60 ASD (ages 2–12y): FMT + RAU (n = 30) vs. RAU (n = 30)	Metronidazole (0.2 g, 3×/d) for 3d pre-FMT	Oral FMT capsules: 50 g feces → saline (1:3) → capsules (4.5 g/d × 6d)	4 weeks	FMT + RAU > RAU in ABC/CARS reduction	FMT + RAU > RAU in GSRS reduction	Altered microbiota diversity and structure	Synergistic effects of FMT and RAU	FMT group: 2 nausea/vomiting, 3 abdominal distension (mild)
Li et al. (2021), China	Open-label; FMT: 40 ASD (ages 3–17y) with GI symptoms; Control: 16 neurotypical children	Bowel cleansing	Oral FMT capsules: 27 oral (freeze-dried capsules): 2 × 10^14^ CFU/w × 4w; FMT: 13 rectal (colonoscopy): single dose ×4w	8 weeks	CARS: 16% reduction; ABC, SRS, SAS improved	GSRS: 35% reduction; DSR improved	α-diversity: NS; β-diversity shifted toward donor; ↓ *Eubacterium coprostanoligene*	↓ 5-HT, GABA; ↑ DA via gut-brain axis	Oral: 3.7% fever/hyperactivity; Rectal: 7.7% hyperactivity
Liu et al. (2024), China	RCT; 60 ASD: FMT (*n* = 30) vs. probiotics (*n* = 30)	Enema care	FMT: 5 mL/kg live bacterial suspension (1–4 transplants over 8w); Probiotics: 3 tablets ×3/d × 8w	8 weeks	FMT > probiotics in CARS, communication, social skills, sensory/cognitive scores	FMT > probiotics in GSRS reduction	N/A	FMT total efficacy > probiotics at 4w/8w	N/A
Chen et al. (2022c), China	Retrospective; FMT: 328 ASD (ages 6.1 ± 3.4y) with GI symptoms	N/A	Oral FMT capsules: Oral cFM capsules (2×/d × 12d/month)	60 months	ABC improved for 48 m; CARS improved for 60 m	BSFS improved for 48 m; GSRS improved for 24 m	N/A	N/A	Abdominal distension (6.4%), nausea (4.3%), vomiting (2.7%), diarrhea (5.5%), fever (4.0%), hyperactivity (7.3%); all mild/moderate
Chen et al. (2022a), China	Multicenter RCT; FMT: 212 ASD (ages 3–13y); Placebo: 106 ASD	N/A	Oral FMT capsules: cFMs capsules (3–6y: 2 × 2/d; >6y: 3 × 2/d) × 12d/month ×4 m	6 months	N/A	N/A	N/A	N/A	N/A
Chen et al. (2020), China	Prospective; FMT: 221 ASD (nasogastric:10; capsules:190; enema:15)	Lactulose (15 mL × 2/d × 3d)	Oral FMT capsules/FMT: ≥10^10^ CFU/transplant; 6-day course + probiotics/prebiotics	5 years	N/A	N/A	N/A	Efficacy: 64.65% (1 m), 64.66% (12 m), 55.32% (24 m), 56.25% (36 m), 59.09% (48 m), 58.3% (60 m)	No severe complications
Zhao et al. (n.d.), China	Open-label; FMT:24 ASD vs. Waitlist:24 ASD	N/A	FMT: Colonoscopy/gastroscopy FMT × 2 (baseline + 2 m)	4 months	CARS: 10.8% reduction (FMT) vs. 0.8% (Waitlist)	GSI improved (FMT)	↓ *Bacteroides fragilis*	N/A	Fever, allergy, nausea (29.2%); mild/transient
Wei et al. (2023), China	Double-blind RCT; FMT:28 ASD (3–9y); Placebo:14; Controls:30	Rifaximin + bowel cleansing	FMT: XBI-061 (live bacteria) vs. placebo; phased dosing over 25w	25 weeks	N/A	N/A	N/A	N/A	N/A
Li et al. (2024a), China	Open-label; FMT:38 ASD (3–14y; 31 with GI symptoms); Control:30	N/A	Oral FMT capsules: Freeze-dried capsules (1 g/kg × 3 courses over 12w)	20 weeks	ABC↓23%, CARS↓10%, SRS↓6%	GSRS↓51% (post-FMT) → 32% (8w follow-up)	↑ *Dorea; ↓ Blautia, Sellimonas*; no α/β-diversity changes	SDSC↓10%	2 vomiting/fever (self-limiting)
Chen et al. (2022b), China	Retrospective; FMT:11 ASD	N/A	Oral FMT capsules/FMT: 6-day oral/transendoscopic course ×2	4 weeks	ABC/CARS reduced	N/A	N/A	1 allergic reaction, 3 hyperactivity; resolved	N/A
Hu et al. (2023), China	Case report; 1 ASD (7y female)	Vancomycin + bowel cleansing	FMT: 80 mL bacterial solution ×5 over 3 m	3 weeks	CARS/SRS ↓	Rectal ulcer healed	↑ Bacteroides, Ruminococcus; ↓ Bifidobacterium, Anaerostipes; SCFA production ↑	ATEC/CHAT-23/CNBS-R2016 improved	N/A
Wang et al. (2024), China	RCT; FMT:19 ASD (4–12y); Placebo:22	Bowel cleansing	Oral FMT capsules: FMT capsules ×5w + rehabilitation	9 weeks	CARS↓8%, SRS↓13.01%, ABC↓ (FMT > placebo)	GSRS↓37.02% (FMT)	N/A	↓ Urinary 5-HIAA	ALT↑ (within normal range)
Hazan et al. (2024), USA	Case report; 19y ASD	Vancomycin pre-treatment	FMT: Cecal infusion of sister’s stool (300 mL)	16 months	CARS↓34.31%; ATEC↓166.67%	↓ Bloating, constipation, diarrhea	↓ *Lactobacillus animalis*, Proteobacteria; ↑ Actinobacteria, Bifidobacterium	Improved sleep, language, aggression	N/A
Qureshi et al. (2020), USA	Open-label; Same cohort as (Kang et al., 2017)	Same as (Kang et al., 2017)	FMT: Same as (Kang et al., 2017)	8 weeks	N/A	N/A	N/A	Plasma metabolites normalized	N/A
Zeng et al. (2023), China	RCT; FMT + RAU:40 ASD vs. RAU:40	N/A	Oral FMT capsules/FMT: Capsules (20 g/d × 3d) or liquid via tube	6 months	FMT + RAU > RAU in ABC/CARS reduction	FMT + RAU > RAU in GSRS reduction	↑ *Firmicutes* (FMT + RAU)	N/A	Nausea/vomiting (7.5%)
Ward et al. (2016), Canada	FMT:9 ASD (2–21y)	Vancomycin	Oral FMT capsules/FMT: Encapsulated stool + enema	N/A	N/A	N/A	Altered Bacteroides, Clostridales, etc.	Younger patients improved more	N/A
Li et al. (2024b), China	FMT:98 ASD (5–9y); Caps (*n* = 73), TET (*n* = 13), NJT (*n* = 12)	Bowel prep for TET	Oral FMT capsules/FMT: Caps:1 g/kg × 3 courses; TET/NJT:3 courses	20 weeks	Caps/NJT > TET in ABC/CARS/SRS reduction	GSRS↓ in all groups	N/A	NJT > Caps/TET in SDSC reduction	5 vomiting, 2 abdominal pain, 2 nausea/fever (mild)
Zhang et al. (2022), China	WMT:49 ASD (3–14y); Constipation:24; Control:25	N/A	WMT: TET/nasojejunal tube (120 mL/d × 6d)	16 weeks	CARS/ABC↓	Constipation improved (BSFS)	N/A	Improved sleep quality	N/A
Liu et al. (2023), China	WMT:24 ASD (>2y)	Lactulose	WMT: TET/nasojejunal tube (120 mL/d × 3d)	16 weeks	ABC↓	Intestinal lesions improved	↑ *Prevotella*_9; ↓ *Bacteroides, Flavonifractor*; ↑ DAO, D-lactate, endotoxin	Improved constipation/sleep	N/A
Pan et al. (2022), China	WMT:42 ASD	N/A	WMT: 5.0 × 10^13^ bacteria/d × 6d via TET	30 weeks	ABC/CARS↓ with course number	Constipation resolved after 4 courses	N/A	SDSC↓; systemic inflammation↓	N/A
Hu et al. (2025), China	WMT:129 ASD	N/A	WMT: 5.0 × 10^13^ bacteria/d × 6d via TET	N/A	ABC/CARS↓	Constipation/diarrhea resolved	N/A	SDSC↓; efficacy ↑ with course number	14 mild AEs (566 courses): fever (1.0%), diarrhea (0.5%), etc.
Zhong et al. (2025), China	Single-center retrospective cohort; 83ASD	N/A	WMT: Donor screening + automated purification (GenFMTer); Washed 3x; 60–90 mL via TET; 6-day/course, 1-month intervals (up to 6 courses)	Jun 2019 - Oct 2022 (follow-up post-WMT)	ABC, CARS, SDSC scores significantly reduced after each WMT course; Improvements sustained with course number	Structural changes in gut microbiota (PCoA); ↓ Bacteroides, ↓ Lachnoclostridium, ↑ Collinsella; No significant alpha diversity change	Tongue-coating: ↑ Rhodococcus, ↑ Pseudomonas, ↓ Capnocytophaga, ↓ Alloprevotella, ↓ Corynebacterium; ↑ Haemophilus (neg. Corr. with CARS); ↓ chemoheterotrophy and fermentation functions	Predictive model using pre-treatment tongue-coating microbiota + clinical features (AUC = 0.73); Performance comparable to gut microbiota model (AUC = 0.75)	7 AEs in 268 courses (fever, diarrhea, abdominal pain, vomiting); All self-limited/resolved with treatment
Zheng et al. (2025), China	Retrospective; 44 ASD (single-donor:17; multi-donor:27)	N/A	WMT: 2 courses of WMT via TET; 6 days/course, 4-week interval	8 weeks	ABC, CARS, SDSC scores ↓ after 2 courses; no difference between single- vs. multi-donor	N/A	↑ α-diversity in effective group; ↑ Lactobacillus in effective group; ↑ Faecalibacterium, Campylobacter, Sphingomonas in ineffective group	No severe adverse reactions	Mild: diarrhea (5), fever (4), abdominal pain (1); all resolved
Pan et al. (2025), China	Retrospective; 103 ASD (non-constipation:59; constipation:44)	N/A	WMT: 2–6 courses of WMT via TET; 6 days/course, 1-month interval	Up to 30 weeks	ABC, CARS, SDSC scores ↓ in both groups; no mid-term difference between groups	BSFS improved in constipation group; D-lactate ↓ in constipation group	↑ α-diversity and ↑ Bifidobacterium in constipation group after 1st course	Sleep improved; stool normalized in constipation group	Mild: fever (2), diarrhea (3), abdominal pain (1)

CARS: Childhood Autism Rating Scale; ABC: Autism Behavior Checklist; SRS: Social Responsiveness Scale. VABS-II: Vineland Adaptive Behavior Scales; GSRS: Gastrointestinal Symptom Rating Scale; DSR: Daily Stool Record. SDSC: Sleep Disturbance Scale for Children; RAU: Routine Autism Rehabilitation; TET: Transendoscopic Enteral Tubing. NJT: Nasojejunal Tube; SCFA: Short-Chain Fatty Acids; 5-HIAA: 5-Hydroxyindoleacetic Acid.

In the simple treatment of ASD with WMT, Pan et al. (2022) found that WMT significantly improved the core symptoms of ASD (ABC and CARS scores decreased), sleep disorders (SDSC scores decreased), and gastrointestinal symptoms (the proportion of constipation decreased, and the shape of stool improved). The number of treatment sessions was positively correlated with the therapeutic effect: as the number of WMT sessions increased (especially within 3 sessions), the ABC and SDSC scores further decreased. Systemic inflammatory indicators (WBC, globulin) also significantly decreased. It was concluded that WMT could significantly improve the symptoms, sleep and gastrointestinal problems of ASD, and that multiple treatments were superior to single treatments. Zhang et al. (2022). found that the SDSC score of the constipation group significantly improved after the second WMT (*p* = 0.026), and sleep quality improved. The improvement in stool shape (BSFS) was synchronous with the improvement in sleep. The non-constipation group did not show a deterioration in sleep, indicating that WMT was safe. It was concluded that WMT had a significant effect on improving sleep in children with ASD accompanied by constipation, and did not cause deterioration of other children’s symptoms. Liu et al. (2023) found that WMT significantly improved sleep disorders and constipation, with decreased ABC and SDSC scores. The bacterial structure changed: *Prevotella_9* was upregulated, and *Bacteroides/Flavonifractor/Parasutterella* were downregulated. Metabolic pathway analysis showed that WMT regulated pathways such as glycolysis, fatty acid *β*-oxidation, and L-1,2-propanediol degradation to reduce toxic metabolites and enhance detoxification ability. It was concluded that WMT improved the sleep and behavioral symptoms of ASD children through regulating the intestinal microbiota and its metabolites. Hu et al. (2025) found that WMT significantly improved the symptoms, sleep disorders and GI symptoms (constipation, diarrhea, abnormal stool shape) of ASD. The 6-session WMT course was the most effective, and the therapeutic effect improved with the increase in the course. Multiple regression analysis showed that children with diarrhea, abnormal stool shape, long disease course, no other treatment, and severe conditions had a better response to WMT. It was concluded that WMT had a significant effect on ASD children, that GI symptoms could be used as a predictive indicator of therapeutic effect, and that multiple treatments could further enhance the therapeutic effect. The benefits of WMT for ASD children from different perspectives such as therapeutic effect, sleep, bacterial metabolism, and GI symptoms were systematically demonstrated, and it was unanimously concluded that: WMT was safe and effective, could significantly improve the core symptoms, sleep disorders and gastrointestinal problems of ASD; the more treatments, the better the effect; at least 3–6 sessions of treatment were recommended; changes in intestinal microbiota structure and metabolites were the potential mechanism; children with GI symptoms (such as constipation, diarrhea) may benefit more. Zheng et al. (2025) found that among 83 ASD patients, the ABC, CARS, and SDSC scores significantly decreased as the WMT treatment progressed. This indicates that WMT can effectively improve the social impairments, repetitive behaviors, emotional problems, and sleep quality of ASD children. During 268 WMT treatments, only 7 minor adverse reactions (such as fever and diarrhea) occurred, and no serious adverse events were observed. After WMT, the intestinal microbiota structure significantly changed. *Bacteroides* and *Lachnoclostridium* (positively correlated with the severity of ASD) significantly decreased. *Collinsella* (a beneficial bacterium) significantly increased. Changes in tongue coating microbiota: *Haemophilus* (negatively correlated with the severity of ASD) significantly increased. *Capnocytophaga*, *Alloprevotella*, and *Corynebacterium* (positively correlated with ASD symptoms) decreased. The tongue coating microbiota structure was more similar to that of typical developing (TD) children. Functional prediction: The functions related to chemosynthetic heterotrophy and fermentation in the tongue coating microbiota significantly decreased after WMT. In the WMT-treated germ-free ASD mice: social behaviors improved and repetitive behaviors were reduced. The levels of Th17 cells in the brain and pro-inflammatory cytokines (such as TNF-*α*, IL-1β, and IL-6) were decreased. The intestinal barrier function was improved (increased villus height and reduced crypt depth). It is indicated that healthy microbiota transplantation can improve ASD behaviors and neuroinflammation by regulating the gut-brain axis. Zheng et al. (2025) found that after two courses of WMT treatment for 44 ASD children, the ABC, CARS, and SDSC scores significantly decreased (*p* < 0.05), and the core symptoms and sleep quality improved. There was no significant difference in efficacy between the single-donor and multi-donor groups after two courses of treatment, although there were differences in ABC and SDSC scores after the first course (*p* = 0.049, *p* = 0.019). The alpha diversity of the intestinal microbiota significantly increased in the effective group, and *Lactobacillus* was enriched in the effective group, while *Faecalibacterium*, *Campylobacter*, and *Sphingomonas* dominated in the ineffective group. It was concluded that single-donor and multi-donor WMT have equivalent efficacy in ASD treatment, supporting the use of the multi-donor strategy in clinical practice to address the issue of donor availability, and emphasizing the correlation between microbiota diversity and efficacy. Pan et al. (2025) found that WMT significantly improved ABC, CARS, and SDSC scores regardless of whether constipation was present (*p* < 0.05). In the constipation group, the ABC score improved more slowly in the first two courses of treatment, but there was no difference with the non-constipation group after the third course. After WMT, the diversity of the intestinal microbiota significantly increased in the constipation group, and the relative abundance of *Bifidobacterium* increased, while the D-lactic acid level decreased, suggesting improvement in intestinal barrier function. It was concluded that constipation does not affect the mid-term efficacy of WMT, and WMT can simultaneously improve ASD core symptoms and constipation problems, especially by promoting the abundance of beneficial bacteria such as Bifidobacterium, promoting intestinal health.

In summary, WMT improves ASD symptoms, enhances gut microbiota diversity, and elevates quality of life, with sustained therapeutic effects. Most studies affirm its safety profile, characterized by mild, reversible adverse events and no severe complications. As a novel strategy, WMT exhibits promising potential for ASD treatment, supported by robust safety and long-term efficacy data. Future research should elucidate WMT’s mechanisms, optimize protocols, and establish standardized workflows to maximize therapeutic outcomes and safety.

#### 4.3.1 Comparison between WMT and FMT

WMT represents a significant technological advancement over traditional FMT. Table 4 provides a structured comparison between WMT and FMT regarding implementation standards, standard operating procedures (SOPs), clinical efficacy, and advantages and disadvantages.

**Table 4:** Comparison between WMT and FMT in ASD treatment.

Aspect	WMT	FMT
Implementation standards and donor screening	Strict, standardized donor screening per Nanjing Consensus (Shi, 2020) Comprehensive evaluation: medical history, blood/feces pathogen tests, mental health assessment Often utilizes standardized donor banks	Similar rigorous screening principles recommended Practice may vary significantly between centers More reliant on individual donor-recipient pairing
Standard operating procedures (SOPs)	Standardized, automated processing: Uses intelligent microbiota separation system (Zhang et al., 2020; Shi, 2020) Multi-step washing: Repeated centrifugation and washing to remove supernatant, effectively eliminating undigested food residues, fungi, parasite eggs, and pro-inflammatory metabolites (Zhang et al., 2020) Quality Control: High, due to automated, closed-system preparation. Final product is a purified bacterial suspension. Preparation Environment: Strictly controlled GMP-grade laboratory	Less standardized, often manual processing: Manual homogenization and filtration (Zhang et al., 2020) Limited purification: Primarily aims to remove large particulate matter. Retains most soluble components and metabolites. Quality Control: Variable, depends on individual center protocols. Preparation Environment: Standard laboratory setting, often open or semi-open procedures
Clinical efficacy and symptom impact	Core ASD Symptoms: Shown to significantly improve scores on ABC (average reduction rate 23.5%, range 15.7–34.31%), CARS, and social responsiveness in clinical studies (Kang et al., 2017; Kang et al., 2019; Pan et al., 2022; Hazan et al., 2024; Zhong et al., 2025) GI Symptoms: Demonstrates marked improvement in constipation, diarrhea, and abdominal pain (average remission rate 72.3%, range 58–89%) (Kang et al., 2017; Zhang et al., 2022; Hu et al., 2025) [115] Other Comorbidities: Improves sleep disorders (SDSC score reduction) and reduces hyperactivity (Zhang et al., 2022; Liu et al., 2023; Pan et al., 2025) Durability: Studies suggest sustained benefits for up to 2–5 years with long-term follow-up (Kang et al., 2019; Chen et al., 2020; Zhong et al., 2025) Positive Outcome Rate: 60–80% of patients show significant improvement in core symptoms (Li et al., 2019; Li et al., 2022; Zheng et al., 2025)	Core ASD Symptoms: Effective in improving behavioral scores (ABC average reduction rate 18.2%, range 10.8–24%) in multiple studies (Sharon et al., 2019; Kang et al., 2017; Zhao et al., n.d.) GI Symptoms: Effective at alleviating GI distress (average remission rate 65.5%, range 50–77%) (Kang et al., 2017; Li et al., 2021) Durability: Benefits observed for up to 2 years, but long-term data beyond 2 years is less common (Kang et al., 2019) Positive Outcome Rate: 55–70% of patients show significant improvement in core symptoms (Kang et al., 2017; Li et al., 2021)
Safety and adverse events	Incidence: Lower incidence of adverse events (AEs) reported (Zhang et al., 2020; Zhang et al., 2022) Common AEs: Mild and transient (e.g., abdominal distension, low-grade fever, transient hyperactivity) (Pan et al., 2022; Hu et al., 2025) Severe AEs: No severe AEs (e.g., systemic infection) reported in ASD clinical studies to date. Reduced risk due to removal of harmful components.	Incidence: Higher incidence of short-term AEs reported (Zhang et al., 2020) Common AEs: Abdominal cramping, diarrhea, nausea, and vomiting are more frequent (Zhang et al., 2020) Severe AEs: Small but potential risk of pathogen transmission if screening fails; theoretical risk of severe infection.
Advantages	Superior Safety Profile: Purification process reduces pro-inflammatory molecules and potential pathogens. Enhanced Patient Acceptance: Clear, odorless bacterial suspension addresses ethical and aesthetic concerns (Shi, 2020). High Standardization and Reproducibility: Automated SOPs ensure consistent product quality and facilitate multi-center applications. Potential for Better Efficacy: Removal of inhibitory substances may allow for better engraftment of beneficial microbiota.	Established History: Longer track record and more extensive clinical experience for recurrent C. difficile infection. Technical Simplicity: Lower technological barrier for preparation (e.g., manual blending). Lower Cost (Per Procedure): Less equipment and processing required.
Disadvantages and challenges	Higher Technical and Infrastructure Demand: Requires specialized equipment (centrifuges, automated systems) and GMP-grade labs. Increased Cost (Initial Setup): Higher initial investment for infrastructure and standardized processing. Limited Long-Term Data: As a newer technique, very long-term (>5 years) safety and efficacy data are still being accumulated.	Safety Concerns: Higher risk of AEs and potential for pathogen transmission due to less purified material. Poor Standardization: Product consistency varies between batches and centers, complicating research and clinical application. Lower Patient Acceptance: The nature of crude fecal material poses significant ethical and aesthetic barriers for some patients.

The positive outcome rate and symptom improvement rate are descriptive statistical results based on the clinical data of the studies included in Table 3.

### 4.4 Mechanisms of WMT in treating ASD

The precise mechanisms by which WMT alleviates autism spectrum disorder (ASD) remain unclear. As an advanced form of FMT, WMT’s therapeutic effects are primarily attributed to the transplantation of a purified, diverse, and functional microbial community, which may exert more potent and targeted modulation of the “microbiota-gut-brain axis” compared to probiotics or conventional FMT. Current hypotheses suggest that gut microbiota and their metabolites may influence the central nervous system through metabolic pathways, vagus nerve activation, and immune modulation, forming a “microbiota-gut-brain axis” that impacts the onset and progression of ASD (Sharon et al., 2016; Cryan et al., 2019).

ASD patients often exhibit metabolic dysregulation, and gut microbiota can regulate gene expression and neuronal function. Studies indicate that microbial-derived short-chain fatty acids (SCFAs), particularly butyrate and propionate, modulate the expression of genes such as FMR1 and Neurexins, potentially improving ASD symptoms (Kang et al., 2017; Qureshi et al., 2020; Hsiao et al., 2013). Gut microbes also synthesize neurotransmitters, including serotonin (5-HT), dopamine, and *γ*-aminobutyric acid (GABA), which directly affect central nervous system activity (Yano et al., 2015). For instance, probiotics may enhance the conversion of tryptophan to serotonin, mitigating neuroinflammation (Fattorusso et al., 2019).

The vagus nerve connects the brain with the gastrointestinal tract. The metabolites of the intestine can activate it and affect the central nervous system, thereby participating in the onset of ASD (Cryan et al., 2019)^.^ 5-hydroxytryptamine and short-chain fatty acids activate the vagus nerve, regulate oxytocin secretion, and improve the social behaviors of ASD (Sgritta et al., 2019). Notably, *Lactobacillus reuteri* can improve the social deficits in ASD mice, possibly related to oxytocin (Sgritta et al., 2019). Moreover, the vagus nerve regulates the function of microglia, and abnormalities in this regulation may lead to ASD phenotypes. The gut microbiota and its metabolites also affect brain function and mood (Erny et al., 2015).

Microorganisms form the intestinal barrier, and their imbalance can lead to reduced permeability, triggering immune activation and inflammation, which may be related to ASD (Vuong and Hsiao, 2017)^.^ The Th17 cells and IL-17 produced by maternal immune activation during pregnancy may increase the risk of ASD in offspring (Cryan et al., 2019; Vuong and Hsiao, 2017). Short-chain fatty acids regulate microglia and participate in neuroinflammation, which may be one of the causes of ASD (Erny et al., 2015; Silva et al., 2020).

WMT, by providing a purified and diverse consortium of beneficial microbes, may more effectively restore gut microbial balance, reduce neuroinflammation, and improve ASD symptoms by targeting these pathways compared to probiotics or conventional FMT. By modulating the microbiota-gut-brain axis, WMT holds promise as a novel therapeutic strategy to address both gastrointestinal and behavioral manifestations of ASD. Future research is needed to fully elucidate these mechanisms and optimize treatment protocols (Fattorusso et al., 2019; Kang et al., 2017).

#### 4.4.1 WMT treats ASD by regulating gut microbiota homeostasis

Probiotics such as Bifidobacterium and Lactobacillus can inhibit the growth of harmful bacteria by modulating intestinal pH. Their surface protein components specifically bind to intestinal epithelial cells, enhancing gut barrier function. Probiotics also stimulate the intestinal immune system, promoting immune cells to secrete the anti-inflammatory cytokine IL-10 while suppressing the pro-inflammatory factor TNF-*α*, thereby alleviating intestinal inflammation and improving ASD symptoms (Fattorusso et al., 2019). In terms of neurotransmitter regulation, probiotics modulate microbial metabolism in the gut, influence tryptophan metabolic pathways, increase serotonin synthesis, and ameliorate neurodevelopmental disorder symptoms (Yano et al., 2015). Prebiotics are selectively utilized by beneficial gut bacteria. Through metabolic activities, these bacteria convert prebiotics into short-chain fatty acids (SCFAs), such as acetate, propionate, and butyrate (Silva et al., 2020). SCFAs provide energy for intestinal epithelial cells, enhance the expression of tight junction proteins, and strengthen the gut barrier (Silva et al., 2020). Additionally, SCFAs act on the brain via the vagus nerve or bloodstream to regulate neurotransmitter levels, improving behavioral symptoms in ASD patients (Cryan et al., 2019; Silva et al., 2020). Synbiotics, combinations of probiotics and prebiotics, exemplify synergistic effects. For instance, synbiotics containing Bifidobacterium and galactooligosaccharides allow prebiotics to nourish probiotics, promoting their colonization and growth. Probiotics regulate immunity and improve gut barrier function, while prebiotic-derived SCFAs amplify these effects, collectively restoring gut microbiota homeostasis and positively impacting ASD symptoms (Fattorusso et al., 2019).

WMT (Microbiota Transplantation) transfers gut microbiota from healthy donors to ASD patients. After colonization, these microbiota reshape the structure and function of the patient’s microbial community. Beneficial bacteria produce SCFAs to suppress harmful bacteria, reducing pathogens and their toxic metabolites like lipopolysaccharides (LPS) (Vuong and Hsiao, 2017). Decreased LPS levels mitigate systemic inflammation and neurological damage (Vuong and Hsiao, 2017). Concurrently, microbiota alterations influence neurotransmitter synthesis and metabolism, modulating neural signaling to alleviate ASD manifestations (Sharon et al., 2016; Yano et al., 2015). Bacteriophages, with host specificity, enable precise targeting of harmful bacteria. For example, phages targeting *Escherichia coli* specifically recognize and lyse these pathogens, restoring microbial balance. Reduced harmful bacteria improve intestinal homeostasis, lower inflammation, and subsequently ameliorate ASD-related symptoms (Vuong and Hsiao, 2017).

#### 4.4.2 Mechanisms of WMT in treating ASD through gut microbiota modulation

**Figure 1:**
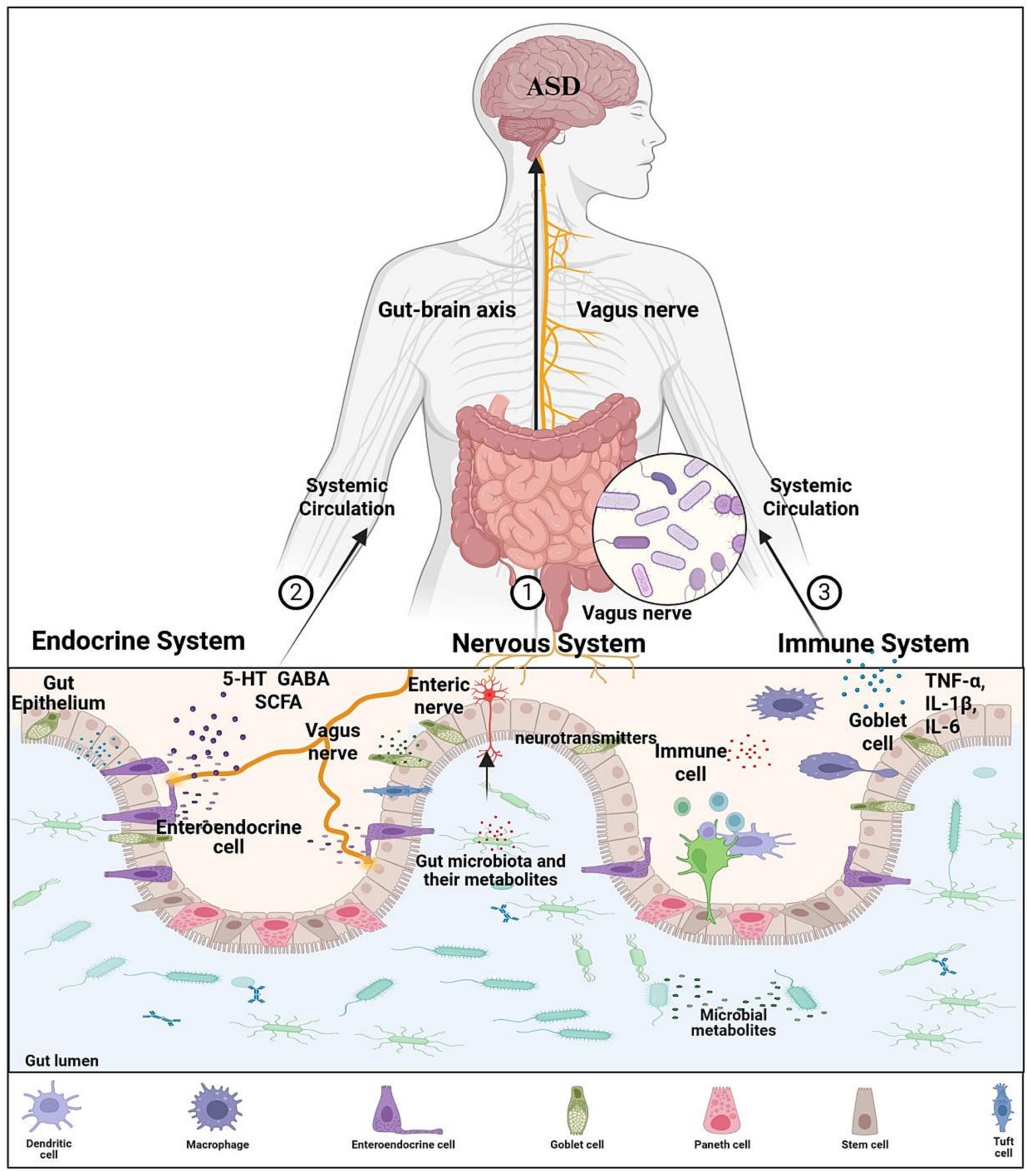
Mechanism of microbiota-gut-brain axis affecting ASD. Gut microbiota and their metabolites can ① regulate the activity of the enteric nervous system, ② promotes production of active substances from intestinal endocrine cells, ③ stimulates the maturation of the intestinal immune system and the release of immune factors. 5-HT, 5-hydroxytryptamine; GABA, gamma-aminobutyric acid; SCFA, short-chain fatty acid; IL-1*β*, Interleukin-1β; IL-6, Interleukin-6; TNF-*α*, tumor necrosis factor-α.

**Figure 2:**
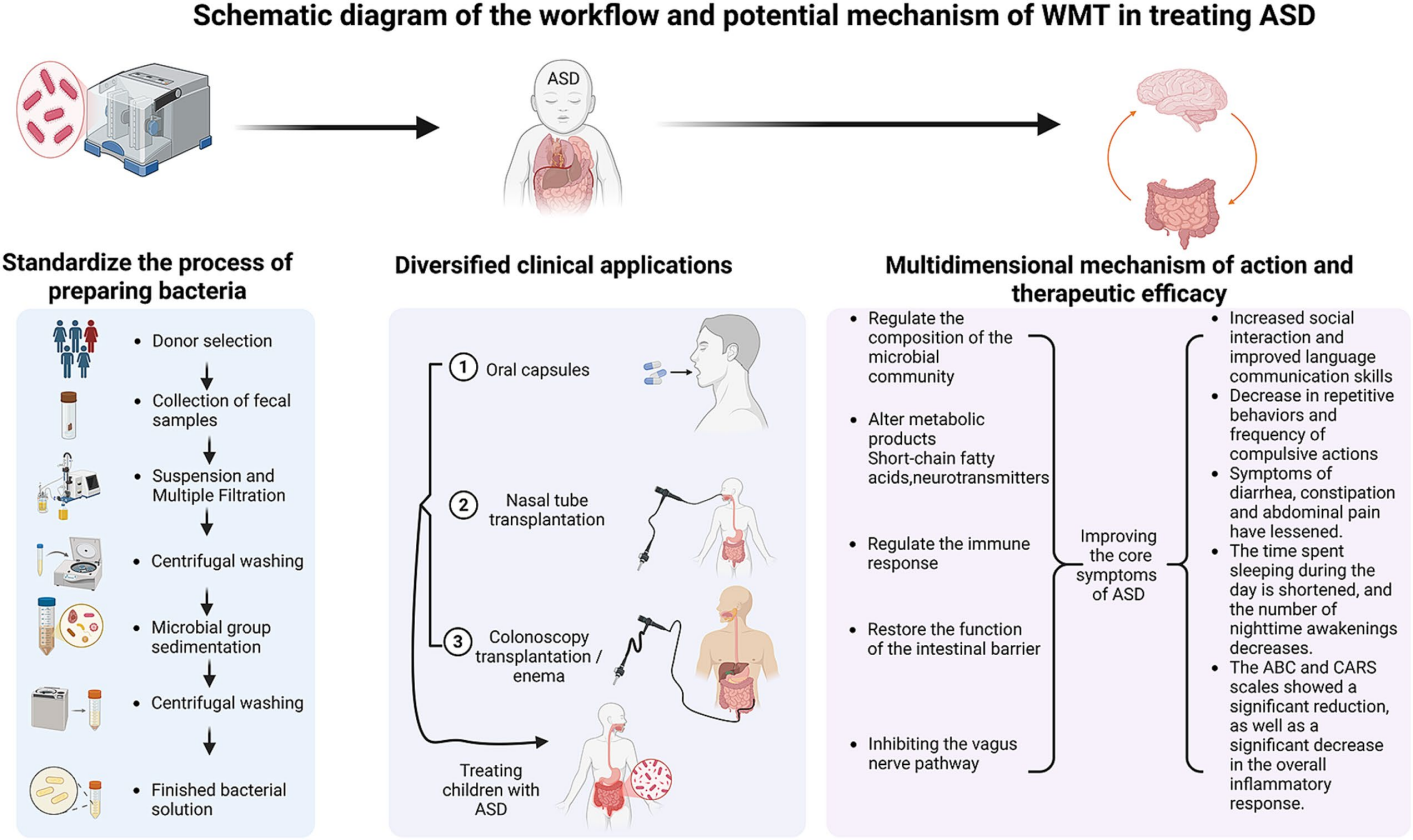
Schematic diagram of the workflow and potential mechanism of WMT in treating ASD. The left panel details the standardized process of preparing the bacterial solution, encompassing critical steps from donor selection and fecal sample collection to suspension, multiple filtration, centrifugal washing, and microbiota sedimentation, culminating in the finished bacterial solution ready for application. The middle panel illustrates the diversified clinical application routes, including oral capsules, nasal tube transplantation, and colonoscopy transplantation/enema, used for treating children with ASD. The right panel summarizes the multidimensional mechanisms of action—including regulating the composition of the microbial community, altering metabolic products (e.g., short-chain fatty acids, neurotransmitters), regulating the immune response, restoring the function of the intestinal barrier, and modulating the vagus nerve pathway—and the consequent therapeutic efficacies observed, such as increased social interaction, decreased repetitive behaviors, alleviated GI symptoms, improved sleep patterns, and significant reductions in ABC/CARS scores and systemic inflammation.

Gut microbiota regulate the expression of tight junction proteins (e.g., ZO-1, Occludin, Claudin) in intestinal epithelial cells. Beneficial bacteria enhance tight junction protein synthesis and assembly via metabolites such as butyrate, strengthening intercellular connections and preventing harmful substances from entering the bloodstream (Sharon et al., 2016; Vuong and Hsiao, 2017). Additionally, gut microbiota stimulate goblet cells to secrete mucins, forming a protective mucus layer that reinforces the intestinal barrier (Sharon et al., 2016). By maintaining barrier integrity, the impact of harmful substances on the nervous system is reduced, alleviating ASD symptoms (Vuong and Hsiao, 2017). Gut microbiota modulate the activity of antioxidant enzymes, including superoxide dismutase (SOD), catalase (CAT), and glutathione peroxidase (GPx). Beneficial bacteria activate signaling pathways in host cells to upregulate antioxidant enzyme gene expression and synthesis. These enzymes scavenge excess reactive oxygen species (ROS), mitigate oxidative stress damage, protect neurons from oxidative injury, and improve neural function, thereby ameliorating ASD-related manifestations (Li and Zhou, 2016). Gut microbiota closely interact with the immune system by activating immune cells in gut-associated lymphoid tissue (GALT). Beneficial bacteria bind to pattern recognition receptors (PRRs, e.g., Toll-like receptors [TLRs]) on immune cells, triggering signaling pathways that induce cytokine secretion (Sharon et al., 2016; Vuong and Hsiao, 2017). Th1 cells release IFN-*γ* to enhance immune defense, while Th2 cells produce IL-4, IL-5, and other cytokines to regulate humoral immunity. This balanced immune response reduces neuroinflammatory disturbances and improves ASD symptoms (Fattorusso et al., 2019; Vuong and Hsiao, 2017). Gut dysbiosis exacerbates inflammation, but microbiota modulation counteracts this. Beneficial bacteria suppress pro-inflammatory factors (e.g., TNF-*α*, IL-6, IL-1β) and promote anti-inflammatory IL-10 secretion (Fattorusso et al., 2019). In inflammatory signaling pathways, lipopolysaccharides (LPS) from harmful bacteria activate the NF-κB pathway, driving cytokine release. Beneficial bacteria and their metabolites inhibit NF-κB activation, block inflammatory signaling cascades, and attenuate both intestinal and systemic inflammation, leading to symptom improvement in ASD patients (Hsiao et al., 2013; Vuong and Hsiao, 2017) (Figure 1).(This revised figure integrates pathways from the original Figures 1, 2, highlighting key mediators like SCFAs, neurotransmitters, immune factors, and the vagus nerve, and indicating how WMT may restore balance).

#### 4.4.3 Gut microbiota-derived metabolites regulate ASD via the gut-brain axis

Gut microbiota influence the synthesis and metabolism of neurotransmitters. For example, specific gut bacteria modulate tryptophan metabolism. After intestinal absorption, tryptophan is preferentially channeled into serotonin synthesis pathways under microbial influence (Yano et al., 2015). Serotonin, a critical neurotransmitter, regulates mood, cognition, and behavior. Elevated serotonin levels improve social interactions and emotional dysregulation in ASD patients (Yano et al., 2015; Li and Zhou, 2016). Additionally, gut microbiota modulate dopamine and *γ*-aminobutyric acid (GABA) metabolism, further impacting neurological function (Sharon et al., 2016; Yano et al., 2015). Fatty acids produced by gut microbiota, such as short-chain fatty acids (SCFAs) and long-chain fatty acids (LCFAs), affect the nervous system through multiple pathways. SCFAs cross the blood–brain barrier via circulation to regulate neurotransmitter levels and neuroplasticity (Silva et al., 2020). Butyrate, a key SCFA, inhibits histone deacetylase (HDAC) activity, altering neuronal gene expression and influencing neurodevelopment (Hsiao et al., 2013; Silva et al., 2020). LCFAs like docosahexaenoic acid (DHA) and eicosapentaenoic acid (EPA) are vital for brain structure and function, improving cognitive and behavioral symptoms in ASD by modulating membrane fluidity and signaling (Li and Zhou, 2016). Specific gut bacteria, such as Bacteroides and Clostridium species, are involved in the synthesis and conversion of LCFAs (Li and Zhou, 2016; Fogelson et al., 2023). Gut microbiota also regulate sphingolipid metabolism, which is critical for neuronal membrane integrity and signaling. Microbial metabolites influence sphingolipid synthesis and degradation pathways. Certain beneficial bacteria enhance sphingolipid production, maintaining neuronal membrane stability and improving synaptic transmission, thereby supporting neurodevelopment and functional recovery in ASD (Li and Zhou, 2016). Certain beneficial bacteria, including Bifidobacterium and Lactobacillus, enhance sphingolipid production, maintaining neuronal membrane stability and improving synaptic transmission, thereby supporting neurodevelopment and functional recovery in ASD (Wu et al., 2023). Beyond these mechanisms, SCFAs activate the vagus nerve to relay signals to the brain (Cryan et al., 2019; Silva et al., 2020). Vagal receptors bind SCFAs, triggering afferent fiber activation that transmits signals to brain regions such as the amygdala and hippocampus, modulating emotion, cognition, and behavior (Cryan et al., 2019; Sgritta et al., 2019). SCFAs also stimulate enteroendocrine cells to secrete hormones like peptide YY (PYY) and glucagon-like peptide-1 (GLP-1), which act systemically to regulate energy metabolism and behavior, alleviating ASD symptoms (Silva et al., 2020). Gut microbiota mediate bile acid metabolism, converting primary bile acids into secondary forms. Beyond aiding fat digestion, bile acids act as signaling molecules via receptors (e.g., FXR, TGR5) to modulate hormone secretion, energy metabolism, and immune responses. Certain bile acids indirectly affect the nervous system by regulating gut barrier function and immune activity, influencing ASD pathogenesis (Vuong and Hsiao, 2017; Li and Zhou, 2016). Gut microbiota modulate branched-chain amino acid (BCAA) metabolism. BCAAs (leucine, isoleucine, valine) play key roles in neuronal metabolism, crossing the blood–brain barrier to support protein synthesis and neurotransmitter pathways. Dysbiosis disrupts BCAA homeostasis, impairing brain function. Restoring microbial balance improves BCAA metabolism, enhancing neurological and behavioral outcomes in ASD (Li and Zhou, 2016). Tryptophan-derived indole compounds, such as indole-3-acetic acid (IAA) and indole-3-propionic acid (IPA), exert anti-inflammatory effects (Yano et al., 2015). IAA modulates gut immunity, reducing neuroinflammatory damage, while IPA activates the aryl hydrocarbon receptor (AhR) to suppress inflammation. Concurrently, serotonin derived from tryptophan metabolism improves ASD behavioral symptoms by optimizing neurotransmitter balance (Yano et al., 2015; Vuong and Hsiao, 2017).

### 4.5 Safety and efficacy of WMT in treating autism

The safety and efficacy of WMT (Microbiota Transplantation) for autism are currently focal points of research. While existing studies on WMT for ASD are limited and involve small sample sizes, preliminary findings suggest its safety and effectiveness, with no significant adverse effects reported. Minor short-term side effects observed post-WMT include abdominal discomfort, diarrhea, constipation, and low-grade fever, which are typically self-limiting or resolve spontaneously without long-term consequences. Some studies hypothesize that these transient reactions may be linked to preexisting immunodeficiency or variations in disease severity among patients (Sharon et al., 2016; Cryan et al., 2019). However, current evidence remains confined to small-scale or case studies. Larger randomized double-blind controlled trials are needed to validate therapeutic outcomes and exclude risks such as fecal microbiota transplantation intolerance, transmission of pathogens, or autoimmune complications. Most ASD patients show no intestinal mucosal damage, indicating relatively high safety for WMT. However, immune dysregulation and microbiota abnormalities in ASD patients suggest potential chronic intestinal inflammation, underscoring the importance of stringent donor screening protocols to mitigate risks (Li et al., 2022). Further research is essential to optimize donor selection, standardize transplantation procedures, and establish long-term safety profiles.

### 4.6 Integrating workflow and multidimensional evidence: a visual synthesis of WMT’s application in ASD

Because of the highly promising application of WMT in ASD, so we have developed a comprehensive schematic (Figure 2) that synthesizes the standardized operational procedure with the observed multidimensional therapeutic outcomes. This integration moves beyond a purely descriptive account, providing a cohesive visual summary that underscores WMT’s potential as a structured and evidence-supported intervention for ASD.

#### 4.6.1 Standardized workflow: from donor to patient

The efficacy and safety of WMT are fundamentally rooted in its standardized and rigorous workflow, as visualized in the left and middle panels of Figure 2. The process begins with the meticulous selection of healthy donors and collection of fecal samples, ensuring a high-quality source material. Subsequent steps—suspension, multiple filtration, centrifugal washing, and microbial sedimentation—conducted using intelligent microbiota separation systems, are crucial for removing undigested residues, fungi, parasite eggs, and pro-inflammatory metabolites. This yields a purified, functional microbiota suspension, defining the core technical advantage of WMT over traditional FMT (Zhang et al., 2020; Shi, 2020). The purified microbiota can then be administered via flexible clinical routes (Figure 2, middle panel), such as oral capsules, nasal tube, or colonoscopy/enema, allowing for personalized treatment strategies based on patient tolerance and clinical presentation (Shi, 2020).

#### 4.6.2 Linking multidimensional mechanisms to clinical efficacy

The right panel of Figure 2 graphically connects the proposed multidimensional mechanisms of WMT with the spectrum of clinical improvements documented in ASD patients. As detailed in previous sections, WMT facilitates the restoration of a healthy gut microbial community. This restored community, in turn, exerts its therapeutic effects through multiple, often interconnected, pathways: by producing beneficial metabolites like SCFAs and neurotransmitters (Kang et al., 2017; Qureshi et al., 2020; Hsiao et al., 2013; Yano et al., 2015; Silva et al., 2020), modulating systemic and neuro-immune responses (Sharon et al., 2016; Fattorusso et al., 2019; Vuong and Hsiao, 2017), restoring the integrity and function of the intestinal barrier (Sharon et al., 2016; Vuong and Hsiao, 2017), and influencing brain function via the vagus nerve (Cryan et al., 2019; Sgritta et al., 2019). The convergence of these mechanisms is reflected in the significant alleviation of core ASD symptoms (e.g., improved social interaction and language communication, decreased repetitive/compulsive behaviors), co-morbid GI distress, sleep disorders, and a measurable reduction in systemic inflammation and standardized behavioral scores (e.g., ABC, CARS) (Kang et al., 2017; Kang et al., 2019; Li et al., 2019; Zhao et al., 2017; Qian, 2024; Zhang et al., 2022; Liu et al., 2023; Pan et al., 2022; Hu et al., 2025). This visual synthesis (Figure 2) thereby encapsulates the journey of WMT from a meticulously prepared biological product to a multifaceted therapy capable of addressing the complex pathophysiology of ASD, providing a clear and compelling framework that supports its application in this field.

#### 4.6.3 Future directions guided by the workflow

The structured workflow presented in Figure 2 also highlights critical junctures for future research and protocol optimization. Efforts can be directed towards refining each step, such as enhancing donor-recipient matching criteria, optimizing the number and timing of treatments (Pan et al., 2022; Hu et al., 2025), and determining the most effective administration route for specific ASD subtypes. Furthermore, establishing correlations between specific microbial or metabolic changes induced by WMT (as suggested in the mechanisms panel) and particular clinical outcomes will be essential for advancing towards personalized microbiota-based therapy for ASD.

## 5 Summary and future perspectives

ASD a neurodevelopmental condition with rising global prevalence, imposes significant burdens on families and society. It is imperative to enhance public awareness, reduce stigma, and strengthen social support for individuals with ASD. Early diagnosis and intervention, multidisciplinary collaboration, public health policies, and increased research investment are critical to improving long-term outcomes. Comprehensive intervention strategies should prioritize resource optimization, long-term evaluation, cognitive enhancement, naturalistic environment training, individualized approaches, skill generalization, integration of emerging technologies, parental collaboration, cost reduction, accessibility, cross-disciplinary innovation, and policy support.

Current ASD treatments remain limited, with pharmacological interventions facing challenges such as disputed efficacy, side effects, and insufficient evidence for long-term safety. TCM attributes ASD pathogenesis to dysfunction of the brain, kidneys, spleen, liver, and heart, advocating herbal medicine and acupuncture. However, TCM research is constrained by low pediatric compliance and unclear mechanistic insights. Notably, TCM’s holistic philosophy aligns with the ecosystem theory of ASD, resonating with emerging therapies like WMT (Microbiota Transplantation), which shows promise in preliminary studies. The integration of its standardized workflow with multidimensional mechanisms and efficacy (Figure 2) provides a tangible and structured framework for its clinical application. Existing evidence suggests WMT is safe and effective for ASD children, though rigorous investigation into its mechanisms and long-term outcomes is needed. Current studies on WMT for ASD are predominantly small-scale or case-based, lacking multicenter, large-sample, high-quality evidence. Randomized double-blind controlled trials are essential to validate efficacy and safety, while addressing risks like donor-recipient incompatibility, pathogen transmission, and autoimmune responses. Although gut dysbiosis is widely observed in ASD, its precise role in pathogenesis remains unclear. Metabolomics, bridging microbial and host interactions, may unlock breakthroughs, yet research on gut microbiota-metabolome relationships in ASD is scarce. Future studies must prioritize linking microbial/metabolomic shifts to symptom improvement to advance biomarker discovery and novel therapies. For WMT optimization, donor-recipient age matching is crucial to minimize immune complications. Parameters such as transplantation dosage, duration, administration routes, antibiotic pretreatment, and standardized evaluation methods require refinement. Mechanistic studies on gut microbiota-neurotransmitter-behavioral interactions are also vital. Long-term follow-up is indispensable for assessing sustained efficacy and safety. While WMT is established for gastrointestinal disorders, its broader application demands robust evidence. Enhancing therapeutic outcomes hinges on selecting donor microbiota with optimal diversity and composition, alongside stringent donor screening to mitigate risks. Future efforts to match optimal donors based on ASD patients’ microbial profiles and clinical subtypes may further enhance therapeutic outcomes. As gut microbiota research advances, WMT holds potential to revolutionize ASD treatment and inspire strategies for other complex disorders. Collaborative innovation, policy advocacy, and sustained investment will be key to translating these insights into transformative clinical solutions.

## 6 Conclusion

This study systematically explores the potential and mechanisms of WMT in treating ASD, with the following key findings: Gut microbiota dysbiosis is a critical pathological feature of ASD; WMT significantly alleviates core ASD symptoms and comorbidities; Multiple mechanisms synergistically regulate the “gut-brain axis”; WMT demonstrates high safety but requires protocol optimization; Future directions focus on precision medicine and multi-omics integration.

WMT offers a safe and effective alternative for ASD patients unresponsive to conventional therapies, reducing familial and societal burdens. It reveals microbiota-metabolite-host interactions, advancing “microbiome-based therapies” for neurological disorders. Standardized protocols and subtype stratification accelerate the translation of laboratory findings into clinical practice, opening new avenues for complex disease treatment. These conclusions deepen the understanding of ASD pathogenesis and provide critical evidence for clinical applications of microbiota-targeted therapies, holding significant scientific and societal value.
